# Why Physical Activity Should Be Considered in Clinical Trials for COVID-19 Vaccines: A Focus on Risk Groups

**DOI:** 10.3390/ijerph19031853

**Published:** 2022-02-07

**Authors:** Miguel Junior Sordi Bortolini, Bernardo Petriz, José Roberto Mineo, Rafael de Oliveira Resende

**Affiliations:** 1 Laboratory of Translational Immunology, Health and Sports Sciences Center, Federal University of Acre, Rio Branco 69920-900, Brazil; miguel.bortolini@ufac.br; 2 Laboratory of Molecular Exercise Physiology, University Center UDF, Brasília 70390-045, Brazil; bernardopetriz@gmail.com; 3 Laboratory of Immunoparasitology “Dr. Mário Endsfeldz Camargo”, Institute of Biomedical Sciences, Federal University of Uberlândia, Uberlândia 38405-317, Brazil; jrmineo@ufu.br; 4 Laboratory on Thymus Research, Oswaldo Cruz Institute, Fiocruz, Rio de Janeiro 21040-900, Brazil; 5 National Institute of Science and Technology on Neuroimmunomodulation (INCT-NIM), Rio de Janeiro 21040-900, Brazil; 6 Laboratory of Allergy and Clinical Immunology, Institute of Biomedical Sciences, Federal University of Uberlândia, Uberlândia 38405-317, Brazil

**Keywords:** physical activity, COVID-19, SARS-CoV-2, vaccine, clinical trials

## Abstract

Since the World Health Organization declared the global COVID-19 state of emergency in early 2020, several vaccine candidates have emerged to control SARS-CoV-2, and some of them have been approved and implemented in vaccination campaigns worldwide. Although clinical trials for these vaccines have been carried out using highly controlled methods with accurate immunological tests, clinical questionnaires did not include questions concerning the physical activity profile among volunteers. It has been well established that physical activity plays a pivotal role in the immune response after vaccination, led by the activation of cytokines, antibodies, and cells. This concept should have been considered when evaluating the efficacy of COVID-19 vaccine candidates, particularly in elderly and obese people. Here, we discuss data from the literature providing strong evidence regarding the importance of analyzing physical activity parameters to improve the accuracy of clinical trials on assessing the efficacy of vaccine candidates.

## 1. Introduction

Vaccines are a vital tool for modern society. Their administration is essential for the prevention of infectious diseases, as they represent the most reasonable cost-effectiveness ratio among global interventions to eradicate diseases [1,2]. It is not by chance that the investigation of mechanisms to prevent diseases, including those caused by lethal pathogens, has led many teams around the world to develop new methods and assessment protocols for vaccines.

The global population has been facing the worst health crisis in the contemporary era since COVID-19 was declared a health emergency by the World Health Organization (WHO) in 2020. Although new technologies have been put in place to develop effective vaccines against SARS-CoV-2 to control the pandemic [3,4,5,6], clinical trials aimed at evaluating their safety and efficacy have been based on laboratory tests that assess seroconversion and immune cell responses to the viral antigens along with clinical questionnaires containing questions regarding the lifestyle and/or health conditions of volunteers.

COVID-19 was first described as severe pneumonia with an undefined etiology when the first cases were reported [7]. Since then, the puzzle of this disease has been partially solved, although some pieces still need to be filled in. Currently, it is known that SARS-CoV-2 can reach human cells via the interaction between the viral spike (S) protein and angiotensin-converting enzyme-2 (ACE2) receptor in association with transmembrane serine-protease-2 (TMPRSS2) receptor in human cells [8]. This interaction allows for viral internalization and replication, triggering a set of inflammatory cells (e.g., macrophages, neutrophils, and T cells) and molecules (e.g., type I IFN, TLR-4, TLR-7, TLR-8, CXCL-9, and CXCL-6) [9]. These can cause clinical symptoms such as cough, myalgia, headache, fever, anosmia, ageusia, and nasal congestion to varying degrees among patients. In some cases, no clinical symptoms are claimed, with no abnormalities in the image diagnosis [10]. In contrast, in other cases, there can be a cytokine storm, which is characterized by an increased expression of tumor necrosis factor alpha (TNF-α), interleukin (IL)-1, and IL-6, responsible for its progression to severe disease [11]. Notably, the worst prognosis is found in people with comorbidities, particularly those who are obese or elderly. As the induction of an inappropriate immune response characterizes these individuals, they are likely to recover from COVID-19 if they have an active lifestyle [12].

On the other hand, social isolation and stress caused by the pandemic have led most people to reduce or withdraw from physical activity, contributing to immune dysregulation [13,14,15,16,17]. A study compared the risk of intensive care unit admission, hospitalization, and mortality between inactive/less-active people and active people [18]. Inactive people had the most significant risk factor for all situations and active people had the lowest. In addition, physical inactivity, advanced age, and a history of organ transplantation were associated with the most substantial risk for severe COVID-19 outcomes. Indeed, a disturbed immune response to SARS-CoV-2 could be minimized if physical activity was maintained during the pandemic.

The development of COVID-19 vaccine candidates during an ongoing global emergency is unprecedented [19]. Basic information on clinical evaluation may not be adequately obtained due to the overall pressure of the pandemic. Until recently, vaccines took years or decades to be applied in vaccination programs from their early phases. Nonetheless, only 10 months after the SARS-CoV-2 structure was solved and the first experimental data were revealed, at least five vaccines were licensed by regulatory agencies worldwide, and mass vaccination was started in many countries to control the pandemic.

Advances in the research on molecular and physiological responses to exercise have given prominence to the field of exercise immunology. In this sense, it is known that physical activity contributes significantly to stimulating crucial components of the immune system [20,21], promoting the host’s defense against parasites [22,23,24], treating cancer [25], and regulating autoimmune and hypersensitivity components [26,27,28]. It has also been reported that physical exercise is essential for vaccine effectiveness [29,30,31,32], which is crucial for the new COVID-19 vaccines. There is strong clinical evidence concerning the association of SARS-CoV-2 infection with an increased risk of intensive care unit admission, hospitalization, and mortality for inactive or less active people compared to active people [18]. Therefore, we believe that populations with a greater propensity for experiencing more severe COVID-19 could benefit from physical activity assessments to identify vaccine efficacy more accurately for those groups. Thus, we summarized the importance of physical activity in assessing the efficacy and effectiveness of COVID-19 vaccines based on immunological principles.

## 2. Physical Activity and Immune Response

The immune response to vaccination is individual, and pathways can be activated in different ways among individuals [33,34,35,36]. This feature has also been discussed for COVID-19 vaccines, particularly in older people, who are more vulnerable and at higher risk of worsening disease [37]. Several factors may influence the immune response to vaccination, including genetics, nutrition, perinatal status, pathogen class, the environment, and behavioral aspects [32]. It is imperative to highlight physical fitness and physical activity among these factors. In a recent study concerning the efficacy of the tetravalent influenza virus vaccine among athletes and non-athletes, CD4+ T cells were found to be 1.5 times higher among athletes than the control group one week after vaccination [38].

Similarly, they found that athletes had higher cytotoxic T-lymphocyte-associated protein 4 (CTLA4) expression and specific-H1N1 and H3N2 neutralizing antibodies. Additionally, influenza virus-specific antibodies were assessed after vaccination in exercised and inactive older people, with higher influenza virus-specific IgM and IgG titers observed in the first group [39]. Moreover, vaccine efficacy was improved in active elderly patients, represented by higher numbers of monocytes and plasma blasts in the peripheral blood, increased expression of genes associated with phagocytosis, and lower levels of CXCL10 and eotaxin chemokines [40].

Remarkably, the skeletal muscle constitutes an endocrine organ [41], known to release more than 10,000 proteins (e.g., IL-6, IL-15, and IL-7 myokines), 90% of which reach the bloodstream when stimulated by acute and chronic exercise stimuli [42,43,44,45]. These myokines are also associated with lymphocyte memory and were shown to be upregulated after strength exercises, which is essential to improving the protective effect of vaccines [46]. It has also been demonstrated that immunosenescence components may be downregulated in people who exercise, with evident improvement in adaptive immunity [47]. In addition, higher concentrations of interferon gamma (IFN-γ) and TNF-α were found along with decreased IL-4 and IL-10 levels after 12 weeks of swimming in a murine model [24].

Adjuvants are essential for stimulating the immune system after vaccination [48], and physical activity could play this functional role in improving vaccine effectiveness [49,50,51,52]. In a randomized controlled clinical trial, subjects underwent a 45 min session of aerobic exercise before influenza vaccination [31], which led to higher antibody titers 20 weeks after vaccination in young women compared to men, who had a higher IFN-γ response 8 weeks after vaccination, suggesting a sex-specific effect of muscle stress on antibody and cytokine responses to vaccination. In a herpes simplex virus type 1 (HSV-1) murine model, IFN release was also enhanced, as well as humoral immunity (IgG2a/IgG1 ratio), after short-term aerobic exercise associated with vaccination [53]. From this perspective, 133 healthy young adults were randomized to an exercise or control task group [49], and each received a full or half dose of pneumococcal vaccine. Before vaccination, the exercise group completed a 15 min arm and shoulder exercise while the control group rested quietly. The exercise group presented increased antibody levels and better responses than those who rested and received a half dose, and similarly for those who received the full dose. Moreover, higher IgG and IgM titers and enhanced cytokine production were found after vaccination with bacterial and viral antigens associated with bouts of exercise, reinforcing physical activity as a potential vaccination adjuvant [54].

An important issue that must be addressed is the effect of acute and chronic exercise on vaccination responses in addition to age and gender. Although in general, antibody titers were shown to be similar between trained and non-trained older people 4 weeks after influenza vaccination, when comparing gender, trained women presented a significantly higher antibody response against H1N1 influenza than men, indicating that moderate acute aerobic exercise elicits a stronger immune response in women compared to men [50]. In contrast, in adult athletes, specific-T cells and neutralizing antibodies increased after vaccination with tetravalent influenza [38], suggesting that a high frequency and high intensity of training enhances vaccination response.

Furthermore, B and T lymphocytes have been related to physical activity. For example, B cell content was shown to increase up to 88% after acute exercise, among which immature cells (CD27^−^CD10^+^ and CD27^−^IgD^−^) were the most prominent, followed by memory (CD27^+^CD38^−^ and CD27^+^IgD^−^) and naïve (CD27^−^CD10^−^ and CD27^−^IgD^+^) B cells, when healthy adult subjects engaged in 30-min continuous cycling [55]. In addition, regular physical exercise, cognitive scores on memory tests, processing speed, attention, verbal fluency, and executive function were associated with increased numbers of circulating naïve B and T cells after 12 months of moderate- to high-intensity aerobic exercise training [56].

The roles of myeloid (mDC) and plasmacytoid (pDC) dendritic cells are well established as the main antigen-presenting cells in the innate immune system [57], and the synergic immunogenicity process has been specifically addressed to both cell lineages [58,59]. Even when mDCs are not directly infected, they can capture exogenous antigens from the infectious agent and present them on MHC class I molecules by a cross-presentation mechanism. Further, the ability of pDC to present antigens aimed at activating CD4+ and CD8+ T cells in addition to cross-presentation by antigen transfer to bystander mDCs mediated by pDC-derived exosomes were also reported [60].

Interestingly, mDCs can be increased after acute exercise, and they can also increase after strenuous/prolonged exercise. In this context, the total number of DCs increases after a bout of exercise, suggesting preferential mobilization of plasmacytoid DCs during exercise. However, pDCs are shown to decrease immediately post-exercise [61]. Although no CD209+ mDC subset was found in the peripheral blood before, during, or after the exercise, CD205− cells were the most responsive among the mDC subsets [62].

Concerning the investigation of mDC maturation in animals with induced immunosuppression subjected to physical activity, the percentage of DCs increased in trained groups with and without immunosuppressants, associated with higher CD80, CD86, and MHC-II expression levels when compared to untrained animals. Moreover, IFN-γ and IL-12 cytokines may be upregulated in exercised mice, with physical exercise suggested as an inducer of differentiation and maturation of DCs, even within an immunosuppressive environment, in association with the Th1 profile [63]. Regarding the Th2 response, in an experimental study based on the OVA-induced asthma model, moderate physical exercise modulated allergic pulmonary inflammation, increasing Treg and M2 recruitment and pDC activation [28].

Exosomes are another topic of significant debate. This vesicular structure derived from an invagination of the plasma membrane is known to mediate cell-to-cell communication in a broad range of tissues. Notably, physical exercise plays an essential role in releasing exosomes, particularly from T cells, monocytes, and antigen-presenting cells [64,65]. Thus, we speculate that the release of exosomes from muscles during exercise could be another way the immune response to microbes, by either natural infection or vaccination, is improved.

Several studies have shown evidence of the critical crosstalk between the gut microbiota and the immune system. Throughout life, the continuous communication between the microbial consortia and the immune cells of the gut mucosa (e.g., dendritic cells and macrophages) modulates the development of the immune system, giving it the ability to differentiate harmless bacteria from pathogens through the recognition of microbe-associated molecular patterns (MAMPS) [66,67]. Furthermore, the possible role of epithelial-derived exosomes in modulating gut microbiota homeostasis through the delivery of antimicrobial agents is also under discussion [68]. Since the modulating effects of exercise on gut microbiota are already recognized [69], as well as their role in muscle exosome release [70], it is essential to consider the hypothesis of a similar stimulation for epithelial and gut microbes.

## 3. COVID-19 Vaccine Candidates and Physical Activity

Vaccine candidates for COVID-19 began to emerge a few months after the onset of the pandemic. The starting point for the development of these vaccines was, in addition to the discovery of SARS-CoV-2 as the causative pathogen, solving the genomic and structural sequence of the virus. After that, some laboratories, supported by biotechnological and pharmaceutical companies, initiated robust efforts based on established platforms, such as the use of attenuated virus and peptide macromolecules, while others exploited technologies not previously used in this context, such as messenger RNA, DNA, and a viral plasmid vector. To evaluate the efficacy of the vaccines, subjects were recruited for clinical trials from different parts of the globe, especially where the numbers of cases remained higher. However, so far, among the leading COVID-19 vaccine clinical trials [71,72,73,74,75,76], none have been concerned with collecting data regarding the current physical fitness or physical practice of people recruited for the studies.

**Figure 1:**
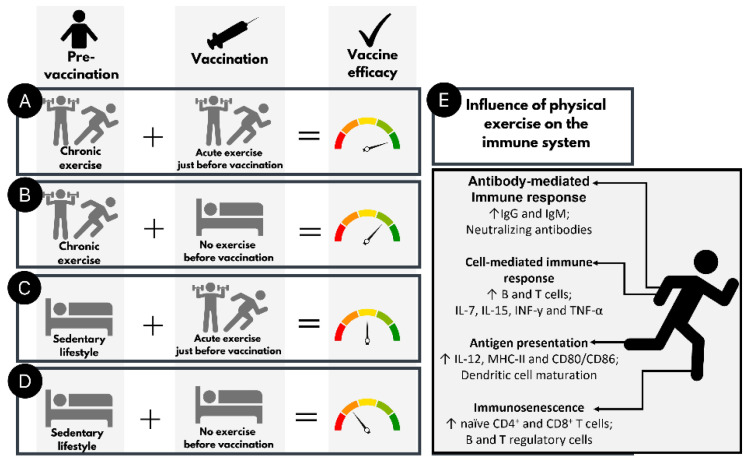
How acute and chronic physical exercise could interfere with immune system and vaccine efficacy, based on published studies. (**A**) subjects who exercise regularly and performed acute exercise just before vaccination; (**B**) subjects who exercise regularly and did not perform acute exercise just before vaccination; (**C**) subjects with a sedentary lifestyle who performed acute exercise just before vaccination; (**D**) subjects with a sedentary lifestyle who did not perform acute exercise just before vaccination. (**E**) List of main influences of physical exercise on immune system. Pre-vaccination is defined as days or months before vaccination; vaccination is defined as the moment of injection; chronic exercise is defined as a regular physical exercise in months or years; acute exercise is defined as moderate/vigorous physical exercise a few minutes or hours before vaccination.

To understand the possible role of physical conditioning and physical activity on the immune response to vaccination, we highlight the main findings regarding the significance of physical exercise before vaccination (Figure 1). A model is presented to compare vaccine efficacy and the subject’s physical condition (Figure 1A–D) based on antibody/cell-mediated cytokine levels, MHC, CD80/CD86 expression, and lymphocyte/dendritic cell proliferation data reported in the literature. When regularly active trained subjects had an exercise session shortly before vaccination, the efficacy was mostly improved (Figure 1A). However, if trained subjects curtailed physical activity just before vaccination, vaccine efficacy could decrease (Figure 1B). When subjects with a sedentary lifestyle exercised a few minutes before vaccination, the efficacy was lower compared to physically active people (Figure 1C) but higher than when they did not perform an exercise before vaccination (Figure 1D). In this context, we hypothesized that active people would be more likely to experience a better immune response than inactive people with most COVID-19 vaccines, and the same would be found for people who exercise just before vaccination.

**Figure 2:**
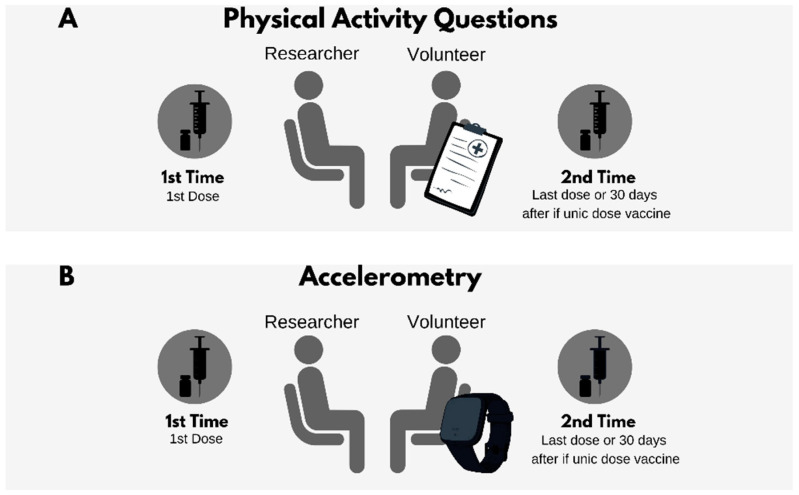
Proposed model for analyzing physical activity level among volunteers with indirect (questionnaire) or direct (accelerometer) method in clinical trials for vaccines. (**A**) Assessment of volunteer’s level of physical activity by questionnaire. Researcher should apply the same questionnaire at least twice: at first dose and same day as last dose. (**B**) Evaluation of physical activity level by accelerometry. Researcher should proceed similarly to questionnaire method. If vaccine is single dose, second assessment of both cases must be carried out 30 days later. Accelerometer assessment requires several days.

To assess the physical activity profile among study participants, we can use indirect or direct tools (Figure 2). Among the former, clinical questionnaires are a good assessment tool, as they allow people’s clinical history to be known using questions that address their physical activity routine. However, this method has some limitations, as it can produce inaccurate results depending on how the questions are answered. In this sense, the use of markers and direct assays (e.g., accelerometry) can provide a more accurate picture of the individual’s physical state in terms of their physical activity practice. In order to provide more accurate representative data, a combination of both forms is also recommended.

## 4. Conclusions

There is evidence that physical activity regulates the inflammatory response. This process is mediated by cellular and humoral immune responses, including distinct cytokine profiles, neutralizing antibody isotypes, and MHC-II and CD80/CD86 expression, leading to increased vaccine efficacy by acting as a potential adjuvant. Due to the global public health emergency scenario, several vaccine candidates have been developed in the short term to control COVID-19 incidence [77,78], and people are currently being vaccinated. In some countries, an administration of a third dose has been authorized [79]. Since physical inactivity and two other factors present the most significant risk for severe outcomes of COVID-19, inactive people, particularly with comorbidities such as obesity, immunosuppression, or immunodeficiency, should be prioritized in third dose vaccination. Although physical exercise has not been evaluated with regard to the COVID-19 vaccines available to date, many potential vaccine candidates are emerging from pharmaceutical companies in Europe, Asia, and Latin America to protect the global population against the spread of the disease. Furthermore, with the emergence of new variants of SARS-CoV-2, the use of a booster dose has been adopted, and those who do not practice regular physical activity, in addition to those who are elderly or immunosuppressed, should be included among the priority groups, as their capacity to produce neutralizing antibodies is considerably reduced. The alternative of developing new vaccine compositions has become clear, and new tests must be conducted to evaluate those formulations. In this regard, as part of the clinical protocol, a complete immunological profile and lifestyle activities should be assessed before and after vaccination, with direct or indirect methods that allow people’s physical activity level to be investigated. People who exercise display robust responses to the vaccines, and this strategy should encourage people to practice physical activity, especially during the COVID-19 pandemic.

## Data Availability

Not applicable.
